# Supplementary material for: Polyamines control inorganic polyphosphate levels during bacterial nitrogen starvation
Source: bioRxiv. 2026 Sep 18:2026.09.16.752086. Preprint. [Version 1] doi: 10.64898/2026.09.16.752086 (PMC13596347; doi:10.64898/2026.09.16.752086)
Supplement: Supplementary file 1 [file media-1.pdf]

SUPPLEMENTAL MATERIAL

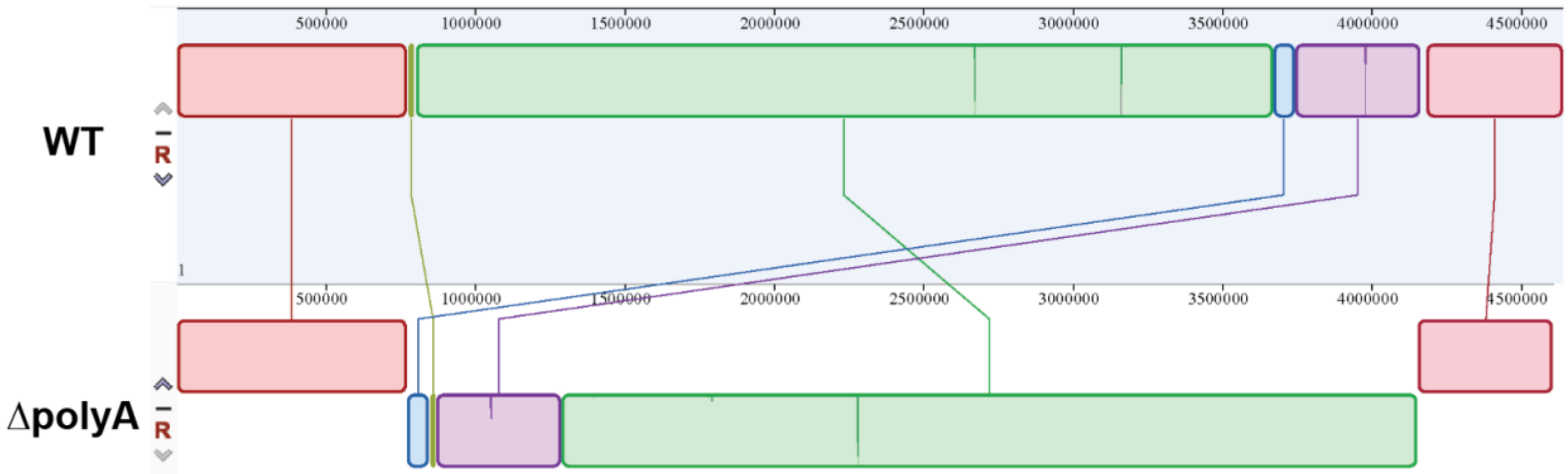

Figure S1. Genomes of indicated *E. coli* MG1655 strains. Chromosomal rearrangements are visualized with Geneious Prime. A 20 kb fragment between *adiA* and *cadA* is lost during Flp-mediated recombination. No essential genes are present in this region.

Table S1 Bacterial strains and plasmids used in this study

**Bacterial strain**		**Marker**	**Source**
E. coli str. K-12 substr. MG1655 (wildtype)			Lab collection
MG1655 Δ*adiA* Δ*cadA* Δ*ldcC* Δ*speAB* Δ*speC* Δ*speDE* Δ*speF* (ΔpolyA)			This study
MG1655 *speDE::Kan*		Kan	This study
MG1655 Δ*cadA ldcC::Kan*		Kan	This study
MG1655 Δ*cadA* Δ*speDE ldcC::Kan*		Kan	This study
MG1655 Δ*speAB* Δ*speC* Δ*speF adiA::Kan*		Kan	This study
MG1655 Δ*ppk*			(1)
MG1655 Δ*adiA* Δ*cadA* Δ*ldcC* Δ*speAB* Δ*speC* Δ*speDE* Δ*speF* *ppk::Kan*		Kan	This study
MG1655 Δ*patA* Δ*puuA tynA::Kan* (ΔPPT)		Kan	This study
MG1655 Δ*adiA* Δ*cadA* Δ*ldcC* Δ*speAB* Δ*speC* Δ*speDE* Δ*speF* Δ*patA* Δ*puuA tynA::Kan*		Kan	This study
MG1655 *ppx::ppx-3×FLAG*		Kan	This study
MG1655 Δ*adiA* Δ*cadA* Δ*ldcC* Δ*speAB* Δ*speC* Δ*speDE* Δ*speF* *ppx::ppx-3×FLAG*		Kan	This study
**Plasmid**	**Description**	**Marker**	**Source**
pSUB11	FLAG tag donor	Kan	(2)
pKD46	λ-red recombinase expression plasmid	Amp	(3)
pCP20	Flp recombinase expression plasmid	Amp	(3)

Table S2 Primers used in this study

Primer	Sequence
adiA-F	GAAGATACTTGCCCGCAACGAAGATTC
adiA-R	GAATCCAGGCGAATTCATCGACAAGCTC
ldcC-F	GTTTGAGCAGGCTATGATTAAGGAAG
ldcC-R	CATCCATCGTCGAGTGGGTGTTGATGAAG
cadA-F	CAGAGCCACTCAATGGATAACCACAC
cadA-R	CATCGAGAGTGGAATACGTATTAGCGAAC
speC-F	GAGCTGGTGACCAGTTTGACCCATATCTCATG
speC-R	CAGGTAATTCAACAGCATGTTCGGAATAC
speF-F	CACGATCGATTTCTCATTCGAGAAATTGAG
speF-R	CTGTTACCTTGATTGACATAATCGACCAGTG
speAB-in-F	CACTTAATAAAATAATTTGAGGTTCGCTCGATTGTGTAGGCTGGAGCTGCTTC
speAB-in-R	TACCCGTGCGCATCGCATCTGGTGCGAATATCCTCCTTAGTTCCTATTC
speAB-out-F	GCGATAGTCGTTAACTGTTTTACACTTAATAAAATAATTTGAGGTTCGCTC
speAB-out-R	CGCATCCGACATTAATGGCACGTTTTACCCGTGCGCATCGCATCTG
speAB-F	CACTGCTGGAAAATCCATGTGCTTATG
speAB-R	CGAACGTAGGTCAGATAAGGCGTTC
speDE-in-F	GTGTTAACAAAGGAGGTATCAACCCCGATTGTGTAGGCTGGAGCTGCTTC

speDE-in-R	GAGCCTGGGAGCTCCGCCAGAGCCGGAATATCCTCCTTAGTTCCTATTCC
speDE-out-F	ATTATGTTGCGCCCTTTTTTTACGGGTGTTAACAAAGGAGGTATCAAC
speDE-out-R	GCAGCAGAAGTAAATAAATCTGGCGGAGCCTGGGAGCTCCGCCAGAG
speDE-F	CTACGTCAAATAATCCCTGATAC
speDE-R	GATGAACTACTACGACGAAGAAAG
patA-F	CATTTGCGCAGCAATCATCAAATC
patA-R	GTGGTGGTCTTATGCAATCTG
puuA-F	GTGGACTAAATTATCGCCATTACTG
puuA-R	TCATCGGCATCATCTCATTTCCTC
tynA-F	GTGACGTTGTCACATTATGCATG
tynA-R	CATACCTTAATTGACGCTAACTTAC
ppx-F-in-F	AGAAAGTACACCAGAAATCGCCGCTGACTACAAAGACCATGACG
ppx-F-in-R	ACATTTCTCGTCGGCCCGCAAAGTACCTCCTTAGTTCCTATTCC
ppx-F-out-F	CTGGCTGGCGGTTGAAAATTGAAGAAGAAAGTACACCAGAAATCG
ppx-F-out-R	GAAAGTGCCTGAATAATGCGGGCCGACATTTCTCGTCGGCCCGCAAAG
ppx-F-seq-F	CAGTTCCTGCCACTGATACAGCTATTG
ppx-F-seq-R	GCCCAGTAGCTGACATTCATC

1. Beaufay F, Amemiya HM, Guan J, Basalla J, Meinen B, Chen ZY, Mitral R, Bardwell JCA, Biteen JS, Vecchiarelli AG, Freddolino PL, Jakob U. 2021. Polyphosphate drives bacterial heterochromatin formation. Science Advances 7.
2. Uzzau S, Figueroa-Bossi N, Rubino S, Bossi L. 2001. Epitope tagging of chromosomal genes in Salmonella. Proceedings of the National Academy of Sciences of the United States of America 98:15264-15269.
3. Datsenko KA, Wanner BL. 2000. One-step inactivation of chromosomal genes in Escherichia coli K-12 using PCR products. Proceedings of the National Academy of Sciences of the United States of America 97:6640-6645.
